# Antioxidant, anticholinesterase and antifatigue effects of *Trichilia catigua* (catuaba)

**DOI:** 10.1186/s12906-018-2222-9

**Published:** 2018-06-05

**Authors:** Nadini Oliveira Martins, Isabella Modelli de Brito, Sandra Syomara O. Araújo, Giuseppina Negri, Elisaldo de Araújo Carlini, Fúlvio Rieli Mendes

**Affiliations:** 1 Departamento de Psicobiologia, UNIFESP, Rua Botucatu, 862, São Paulo, SP CEP 04023-062 Brazil; 2 Centro de Ciências Naturais e Humanas, Universidade Federal do ABC, Rua Arcturus, 03, São Bernardo do Campo, SP CEP 09210-180 Brazil; 3 Departamento de Medicina Preventiva, UNIFESP, Rua Botucatu, 740, 4° andar, São Paulo, SP CEP 04023-900 Brazil

**Keywords:** *Trichilia catigua*, Adaptogen, Antifatigue, Acetylcholinesterase inhibition, Antioxidant, Phenylpropanoids, Cinchonains, Procyanidins

## Abstract

**Background:**

*Trichilia catigua* A. Juss. (Meliaceae) is a species known as catuaba and used in folk medicine for the treatment of fatigue, stress, impotence and memory deficit. The main phytochemical compounds identified in the barks of *T. catigua* are flavalignans, flavan-3-ols and flavonoids which are associated with its antioxidant activity. Pre-clinical studies with *T. catigua* extracts have identified many pharmacological properties, such as anti-inflammatory, antidepressant, antinociceptive, pro-memory and neuroprotective against ischemia and oxidative stress. This study was designed in order to compare the chemical composition and in vitro antioxidant and anticholinesterase activity of four different polarity extracts and selected the one most active for in vivo studies in rodent models of stress, fatigue and memory.

**Methods:**

Hexane, chloroform, hydroalcoholic and aqueous extracts from bark of *Trichilia catigua* were analyzed by RPHPLC-DAD-ESI-MS/MS. Antioxidant activity was assessed by 2,2-diphenyl-1-picryl hydrazyl (DPPH) assay and acetylcholinesterase inhibition by Ellman’s modified method. In vivo studies (stress, fatigue and memory) were carried out with adult male mice and rats treated with hydroalcoholic extract in doses of 25–300 mg/kg (p.o.).

**Results:**

We confirmed the presence of cinchonain IIa, Ia and Ib, as main constituents in the four extracts, while procyanidins were detected only in hydroalcoholic extract. Antioxidant and anticholinesterase activity were observed for all extracts, with most potent activity found on the hydroalcoholic extract (EC_50_ = 43 μg/mL and IC_50_ = 142 μg/mL for DPPH scavenger and acetylcholinesterase inhibition, respectively). The treatment of laboratory animals with hydroalcoholic extract did not protect rats from cold immobilization stress and did not prevent the scopolamine-induced amnesia in mice. However, the treatment of mice with the hydroalcoholic extract partially reduced the fatigue induced by treadmill, since the highest dose increased the spontaneous locomotor activity and reduced the deficit on grip strength after the forced exercise (*p* < 0.05), in some observation times.

**Conclusions:**

These data suggest the hydroalcoholic extract as the most suitable for plant extraction and partially support the folk use of *T. catigua* as antifatigue drug.

**Graphical abstract:**

.

**Figure:**
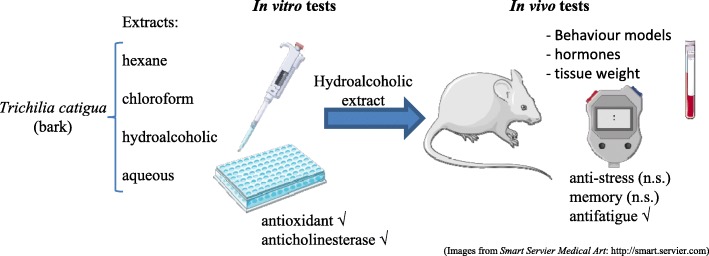
*Trichilia catigua* hydroalcoholic extract exhibits antioxidant and anticholinesterase activity in vitro and reduces the fatigue induced by forced exercise.

**Electronic supplementary material:**

The online version of this article (10.1186/s12906-018-2222-9) contains supplementary material, which is available to authorized users.

## Background

There are several examples of plants used to keep a good health status and to reduce the cognitive deficits that result from aging, such as memory deficit, fatigue and general weakness. Guarana (*Paullinia cupana* Kunth), muirapuama (*Ptychopetalum olacoides* Benth.), nó-de-cachorro (*Heteropterys tomentosa* A. Juss.), damiana (*Turnera diffusa* Willd. ex Schult.) and catuaba (*Trichilia catigua* A. Juss.) are species widely used for these purposes in Brazil [1]. The literature has many examples of active principles with remarkable antioxidant action, especially polyphenols. The cholinergic system and the enzyme acetylcholinesterase (AChE) are other important targets for nootropics and cognitive enhancing drugs. In fact, the inhibition of AchE was described for muirapuama and guarana [2, 3], two Brazilian species used for the improvement of cognitive functions similarly to the folk use of *Trichilia catigua*.

*Trichilia catigua* (Meliaceae) is a species of South America, known as catuaba, tatuaba and catiguá, and used in folk medicine as a tonic for the treatment of fatigue, stress, impotence and memory deficit [1, 4–6]. These popular uses are typical of an adaptogen, which is supposed to decrease the consequences of stress and improve physical and cognitive performances both in healthy and ill patients [1]. The most common popular form of preparation is as “garrafada” (the maceration of the barks in alcoholic drinks, usually 38–48% alcohol). Several other species are also known as catuaba and are used for similar purposes, but most of the available commercial products use barks of *T. catigua* [4].

Flavonoids, tannins, alkaloids, saponins, among other phytochemical classes were identified in the barks of *T. catigua* [4]. The bark contains high concentrations of polyphenols including flavan-3-ols (procyanidin B2, epicatechin, catechin), flavalignans (cinchonains Ia, Ib, IIa, IIb) and phenylpropanoid derivatives (chlorogenic acid) [7–10]. The main constituents of *T. catigua* exhibited potent antioxidant activity, which is important in the prevention of cellular damage triggered by oxidative stress in acute and chronic neuropathological conditions [6].

Pre-clinical studies with *T. catigua* extracts have shown many pharmacological properties, such as anti-inflammatory [11], antinociceptive [12], antidepressant [5, 13, 14], pro-memory [5] and neuroprotective against ischemia and oxidative stress [6, 15, 16]. The antinociceptive and antidepressant effects are attributed mainly to dopaminergic action [12, 13] and were also described for a commercial preparation containing *T. catigua*, *Paullinia cupana*, *Ptychopetalum olacoides* and *Zingiber officinale* Roscoe [4].

Even though many biological activities have been reported to *T. catigua*, its adaptogen-like effect was not fully evaluated. Thus, the present study was designed in order to compare the chemical composition and in vitro antioxidant and anticholinesterase activity of four extracts with different polarity and select the one most active for in vivo studies in rodent models of stress, fatigue and memory.

## Methods

### Plant material and extracts preparation

Ground barks of *T. catigua* were obtained from Santos Flora with quality control assurance. The extracts were prepared using 10% of botanical material in PA grade solvents (Synth, Diadema, Brazil). The aqueous extract was prepared by decoction (50 g of plant in 500 mL of boiling water); the hydroalcoholic extract was prepared by turbolysis (100 g of barks in 1 L of ethanol: water 50% under vigorous agitation); the chloroform and n-hexane extracts were prepared by macerating 25 g of plant with 250 mL of solvent for four days at room temperature, followed by 50 min in ultrasound. The extracts were filtered, concentrated in a rota-evaporator and then dried in a fume hood (chloroform and hexane extracts) or lyophilized (aqueous and hydroalcoholic extracts). The percent yields of extractions were 15.25 (hydroalcoholic), 13.52 (aqueous), 1.98 (chloroform) and 1.76 (hexane). All extracts were analyzed by HPLC-DAD-ESI-MS/MS in order to obtain their respective phytochemical profile.

### Phytochemical analysis

#### Thin-layer chromatography (TLC)

The four extracts were examined by TLC using silica gel plates (200 μm layer thickness, Merck). The extracts were dissolved in a mixture of methanol and chloroform (1:1) and the TLC was developed with chloroform: methanol:water (65:35:10, *v*/v/v) as the mobile phase. The plates were visualized by UV at 254 and 365 nm and by spraying with a 5% vanillin solution in 10% HCl in ethanol (Synth, Diadema, Brazil) (*v*/v), followed by heating the plate. Flavanols (condensed tannins, monomers, dimers) react with vanillin in acidic medium to yield a red adduct. Compounds were also revealed by spraying 1% ethanolic FeCl_3_ solution (Synth, Diadema, Brazil).

#### RPHPLC-DAD-ESI-MS/MS analyses

The RPHPLC-DAD-ESI-MS/MS ion trap analysis was conducted in the DADSPD-M10AVP Shimadzu system equipped with a photodiode array detector coupled to Amazon Speed ETD, Bruker Daltonics, which consisted of two LC-20 AD pumps, SPD-20A diode array detector, CTO-20A column oven and SIL 20 AC auto injector (Shimadzu Corporation, Kyoto, Japan). The mass detector was a quadrupole ion trap equipped with atmospheric pressure ionization source through electrospray ionization interface, which was operated in the full scan MS/MS mode. All the operations, acquisitions and data analysis were controlled by the Shimadzu CBM-20A system controller. HPLC grade water was prepared with distilled water using a Milli-Q system (Millipore, Waters, Milford, MA, USA).

The extracts (3.33 mg/mL) were dissolved in a mixture of water milli-Q and methanol (1:1), filtered by a 0.45 μm PFTE filter and then an aliquot of 30 μL was injected into the HPLC system. Spectral UV data from all peaks were collected at the range 240–400 nm and chromatograms of flavanols were recorded at 280 nm. Separation of the mixture of the constituents was performed in reverse phase Luna Phenomenex – C18 RP-18 column (4.6 × 250 mm, 5 μm, Hewlett Packard) connected to a guard column. The mobile phase was composed by eluent A (0.1% aq. formic acid) and eluent B (methanol) (Merck, Darmstadt, Germany) at the constant flow rate 1.0 mL/min and constant temperature of the oven at 40 °C. The following elution program was used: 0 min – (20% B), 10 min – (30% B), 20 min – (50% B), 30 min – (70% B), 40 min– (90% B), 45 min – (40% B), and finally returned to the initial conditions (20% B) to re-equilibrate the column prior to the next run.

The parameters were set as follows: electrospray voltage of the ion source at − 38 V, capillary voltage at 4500 V, end plate set at 500 V and capillary temperature of 300 °C. Helium was used as the collision gas and nitrogen as the nebulizing gas. Nebulization was added with coaxial nitrogen sheath gas at the pressure of 40 psi. Desolvation was facilitated using a counter-current nitrogen (dry gas) flow set at 9.0 L/min. The spectra were acquired over a mass-to-charge (m/z) ranging between 100 and 1200 mass units with resolution set at 30,000 using the normal scan rate tube lens − 110.0 V. The constituents were fragmented using the auto MS/MS mode. All collision-induced dissociation mass spectra were obtained using helium as the collision gas at the fragmentation voltage from 0.5 up to 1.3 V. Each generated mass spectrum was based on an average of 10 scans. The proposed structure was based on the characteristic fragmentation patterns and comparison with MS data reported in previous studies with the species [17–20] and also by searching the following mass spectral databases: SciFinder Scholar [21], Phenol-Explorer [22], ChemSpider [23] and HMDB [24].

HR-ESI-TOF-MS (high resolution electrospray ionization-time-of-flight-mass spectroscopy) analyses were carried out to determine the exact molecular mass and identify the elemental composition of constituents. The Q-ToF spectrometer MAXIS 3G – Bruker Daltonics consisted of ESI operating at 4500 V, nebulization with nitrogen at 4 Bar and dry gas flow of 8 L/min at temperature of 200 °C. Separation of the constituents was performed in reverse phase Luna Phenomenex – C18 RP-18 column (4.6 × 250 mm, 5 μm, Hewlett Packard) connected to a guard column and a mobile phase composed by eluent A (0.1% aq. formic acid) and eluent B (methanol) at the constant flow rate 1.0 mL/min and constant temperature of the oven at 40 °C. The same chromatography conditions were used in the analyses by Q-ToF. Calculations were performed using the high precision calibration quadratic algorithm.

### In vitro tests

#### DPPH assay

The test was based on the protocol of Duarte-Almeida et al. [25], with some modifications. An ethanolic solution of 2,2-diphenyl-1-picryl hydrazyl (DPPH) (Sigma, St. Louis, MO, USA) was prepared in order to produce an absorbance between 0.8 and 0.99 at 517 nm. One hundred microliters of each extract diluted in ethanol (Synth, Diadema, Brazil) at initial concentrations of 0.004, 0.01, 0.04, 0.1, 0.4 and 1.0 mg/mL were pipetted in a cuvette and after the addition of 900 μL of DPPH the cuvette was placed in a spectrophotometer (PG Instruments LTD, Leicestershire, United Kingdom) and read during 2 min at 517 nm. Ethanol (vehicle) was used as control and rutin (Acros Organics, New Jersey, USA) as positive control. The percentage of DPPH scavenger (S) was calculated by the formula: S_DPPH_ = [(A_c_ - A_s_)/A_c_] × 100, where A_c_ = absorbance for the control and A_s_ = absorbance for the sample. The concentration of each extract that quenches 50% of DPPH (EC_50_) was calculated by linear regression (% of scavenge vs final concentration) using the mean of 4 assays.

#### Acetylcholinesterase activity

The inhibition of AChE activity was determined spectrophotometrically based on Ellman’s method, as previously reported by Padilla et al. [26], with minor modifications. In microplate, 20 μL of *T. catigua* extracts at different concentrations (0.125, 0.25, 0.5, 1.0, 2.0 and 4.0 mg/mL), 10 μL of acetylcholinesterase (1 U/mL) (Sigma, St. Louis, MO, USA) and 160 μL of 5,5-dithiobis-2-nitrobenzoic acid (DTNB, Ellman’s reagent) 0.33 mM (Sigma, St. Louis, MO, USA) in phosphate buffer (0.1 M, pH 8.0) were pipetted in triplicate and incubated at room temperature for 10 min. Then, 10 μL of acetyltiocholine iodide (20 mM) (Sigma, St. Louis, MO, USA) was added and the plate was immediately placed in a microplate reader (BioTek Instruments, Inc., Winooski, VT, USA). The hydrolysis of acetyltiocholine iodide leads to production of acetic acid and thiocholine, that reacts with DTNB producing the anion 5-thio-2-nitrobenzoic acid, which was monitored at 412 nm during 20 min. Rivastigmine (Exelon®) from Novartis Farmacêutica SA (Barberà del Vallès, Spain) was used as a positive control. The percentage of inhibition (I) of AChE was calculated by the formula: I_AChE_ = [(A_c_ - A_s_)/A_c_] × 100, where A_c_ = absorbance for the control and A_s_ = absorbance for the sample. The concentration of each extract that inhibits 50% of AChE activity (IC_50_) was calculated by linear regression (% of inhibition vs final concentration) using the mean of 3–4 assays.

### Behavioral studies with the hydroalcoholic extract

#### Animals

Male albino Swiss mice (30-50 g) and male Wistar rats (300-450 g), 3–4 months old, from our vivarium (Department of Psychobiology, UNIFESP) were housed in rooms with 12 h light-dark circle, controlled temperature (21 ± 2 °C), with filtered tap water and food (Nuvilab, Brazil) ad libitum, except during the experiments. The animals were kept in in polypropylene cages (4–5 animals each) with pine shavings as bedding material. The animals were randomly divided into the different groups and were treated by gavage (oral administration, p.o.) with water (controls) or hydroalcoholic extract dissolved in water, receiving 0.1 mL per 10 g body weight (mice) or 0.1 mL/100 g (rats). The animals were euthanized in CO_2_ chamber or by decapitation (in the case of stress by immobilization test). The protocols followed the International Guiding Principles for Biomedical Research Involving Animals and were approved by UNIFESP ethics committee (Comissão de Ética no Uso de Animais, from Universidade Federal de São Paulo, São Paulo, Brazil) - protocol #0752/07.

#### Evaluation of motor activity

The effect of the hydroalcoholic extract of *T. catigua* at doses of 50 and 500 mg/kg (p.o.) on animals’ motor activity was evaluated on the rotarod and in activity cages in order to check whether the extracts induce any incoordination or locomotor alteration in high doses. Groups of 10 mice each were placed in plexiglas cages measuring 47.5 cm × 25.7 cm × 20.5 cm equipped with 16 pairs of photoelectric beams distributed in the horizontal axis (Opto-Varimex, Columbus Instruments, Columbus, OH) and the ambulation was detected by subsequent interruptions of adjacent photo beams every 30 min for a total of 120 min, as described by Bezerra et al. [27]. The motor coordination was evaluated in a rotarod apparatus (AVS Projects, São Paulo, Brazil) at 12 RPM before the treatment (basal) and 30, 60 and 120 min after the administration. We used mice pre-selected 24 h before by eliminating those that could not stay on the bar for at least 60s [27].

#### Stress by immobilization and cold

Groups of 9–10 rats were orally treated with *T. catigua* hydroalcoholic extract (25 and 250 mg/kg) or water (stressed control) for 14 days and the stress protocol began on the eighth day. The animals were restricted inside acrylic animal restrainer for 2 h in the morning (8-10 h) and later placed in a cold chamber (10–13 °C) for 2 h in the afternoon (16-18 h) from the 8th to 13th day. The animals were then fasted for 20 h and on the 14th day they were immobilized in a wire screen and placed in the cold chamber for 2 h as described by Mendes et al. [28]. After that, the animals were killed by decapitation and their blood collected for plasma measurement of adrenocorticotropic hormone (ACTH) in a clinical analysis laboratory, and corticosterone by radioimmunoassay according to the manufacturer’s instructions (MP Biomedicals, Santa Ana, CA, USA). The stomachs were immediately removed and the degree and index of ulceration were evaluated according to the scales previously described [28]. Simultaneously, the adrenal glands, thymus and spleen were dissected and weighted in an analytical balance scale. An extra group of rats not subjected to the stress (non-stressed control) received water for the same period, and was used to obtain the normal levels of hormones and tissue weights.

#### Forced treadmill exercise

The physical resistance and fatigue were evaluated in an Exer 3/6 treadmill (Columbus Instruments, Columbus, OH, USA) using the following protocol: 3 min running at 5 m/min for warm-up, and then increasing the speed 3 m/min each minute until 20 m/min. After that, the speed was increased in 2 m/min each minute until reach 26 m/min, and then increased in 1 m/min each minute. The animal was considered exhausted when it refused to run even when challenged with tactile stimuli [28]. Mice that failed to reach the speed of 24 m/min were discarded from the study.

After the basal evaluation, the animals were divided in groups (*n* = 8–10) with similar performance and orally treated with *T. catigua* hydroalcoholic extract (25, 100 and 250 mg/kg) or water (exercised control) for 7 weeks and submitted to the treadmill at the 3rd and 7th week (days 21 and 49) when the maximum speed was registered for each animal. Immediately after reaching exhaustion, each mouse was removed from the apparatus and 25 μL of blood was collect from its tail for lactate quantification in a L-lactate analyzer (YSI 2300 Stat Plus, YSI Life Science, Yellow Springs, OH, USA). After that, the animal was submitted to the grip strength meter (AVS Projects, São Paulo, Brazil) and then placed in a plexiglas cage for measurement of the spontaneous locomotor activity for 1 h, as previously described. The grip strength was measured before (basal) and after the exercise (post-fatigue) in order to determine how the forced exercise affect the strength of each animal. The protocol consisted in placing the animal on a grid and measuring the resistance presented by the mice when it was pulled by the tail, recording the highest figure of three measurements [28]. An extra group of animals treated with water not submitted to the treadmill (non-exercised control) was used to measure the lactate normal level and spontaneous locomotor activity.

#### Classical fear conditioning

Groups of 9–10 mice were orally treated with *T. catigua* hydroalcoholic extract (50 and 300 mg/kg) or water (controls) for 3 weeks (21 days). On the training day, the animals (except the negative control group) received scopolamine (2 mg/kg, i.p. - Sigma, St. Louis, MO, USA) in 0.9% saline 30 min after the extract treatment and 30 min later were placed individually in the context cage dotted with some visual cues. After 2 min of habituation, three electrical shocks (0.3 mA, 1 s duration and interval of 10s) were delivered to the paws of the animal (adapted from Soeiro et al.) [29]. The mice were returned to their home cages and after 24 h placed in the context cage again, without delivery any shock, being the freezing time registered during 5 min.

#### Statistical analysis

The statistical analysis was carried out using the Statistica® and Graph Pad Prism® software. The EC_50_ for DPPH and IC_50_ for acetylcholinesterase were calculated by linear regression using the mean of scavenge or inhibition for each concentration of the tested drugs. ANOVA followed by Duncan post-hoc test was used for parametric analysis. The Kruskall Wallis test followed by the Mann-Whitney test was used to compare the groups on rotarod test due to non-parametric distribution. We adopted a *p* value of 0.05 as statistically significant.

## Results

### Phytochemical analysis

Vanillin reacted with flavanols to yield a red adduct, while the reaction with ferric chloride in TLC developed a brownish grey color, both characteristic for flavan-3-ols, confirming the presence of these constituents, which was corroborated by the ultraviolet spectra of constituents with maximum absorption at 278 nm in the HPLC-DAD analyses.

Table 1 summarizes the mass spectral (MS) data obtained in HPLC-ESI-MS/MS analyses of hexane, chloroform, hydroalcoholic and aqueous extracts from bark of *Trichilia catigua*. The mass spectral data obtained by high resolution tandem mass spectrometry is described in discussion. The identification of constituents was achieved based on the MS data obtained and taking into account the compounds and MS data previously reported for this species by several authors, as detailed in the discussion. As can be seen in Table 1, cinchonain IIa (**4**), cinchonain Ia (**7**) and cinchonain Ib (**12**) were detected in the four extracts, including the apolar hexane and chloroform extracts. Apocynin E (**5**), cinchonain IIa glucoside (**9**) and cinchonain IIb glucoside (**10**) were detected only in chloroform extract, while the procyanidin B2–8-C-rhamnoside (**2**) and procyanidin B2 - (epi)-catechin – (epi)-catechin (**3**) were detected only in hydroalcoholic extract. In the aqueous extract were detected the same constituents found in hydroalcoholic extract, except procyanidins. The HPLC-ESI-MS/MS spectra of the compounds **1**–**12** are provided as Additional file 1.

**Table 1:** Summary of the MS data obtained in the analysis of the hexane (HEX), chloroform (CLO), hydroalcoholic (HA) and aqueous (AQ) extracts from barks of *Trichilia catigua* through HPLC-DAD-ESI-MS/MS

	Rt	HPLC/(−)ESI-MS/MS *m/z* (%base peak)	Proposed structure	HEX	CLO	HA	AQ
**1**	4.0	[M – H]^−^ - 341 MS/MS - 179	6-O-caffeoyl glucoside	–	X	X	X
**2**	14.1	[M – H]^−^ - 723 MS/MS - 597 (20), 433 (80) 425 (90), 407 (100), 289 (20)	procyanidin B2–8-C-rhamnoside	–	–	X	–
**3**	15.3	[M – H]^−^ - 577 MS/MS - 559 (20), 451 (30), 425 (100), 407 (80), 289 (20)	procyanidin B2 (epi)-catechin – (epi)-catechin	–	–	X	–
**4**	17.2	[M – H]^−^ - 739 MS/MS - 587 (100), 451 (40)	cinchonain IIa	X	X	X	X
**5**	18.0	[M – H]^−^ - 467 MS/MS - 449	apocynin E	–	X	–	–
**6**	19.0	[M – H]^−^ - 353 MS/MS - 191	3-O-cafeoylquinic acid	–	–	–	X
**7**	19.6	[M – H]^−^ - 451 MS/MS - 341	cinchonain Ia	X	X	X	X
**8**	21.4	[M – H]^−^ - 613 MS/MS - 503 (100), 451 (50), 393 (20), 341 (20)	bis-(3,4-dihydroxyphenylpropanoid)-substituted catechin	–	X	X	X
**9**	22.2	[M – H]^−^ - 901 MS/MS - 791 (100), 597 (70), 451 (60), 341 (20)	cinchonain IIa glucoside	–	X	–	–
**10**	24.4	[M – H]^−^ - 901 MS/MS - 791 (60), 597 (100), 451 (40), 341 (50)	cinchonain IIb glucoside	–	X	–	–
**11**	24.8	[M – H]^−^ - 613 MS/MS - 503 (100), 451 (10), 341 (10)	cinchonain Id-7- glucoside	–	–	X	X
**12**	26.4	[M – H]^−^ - 451 MS/MS - 341	cinchonain Ib	X	X	X	X

### DPPH assay

The four extracts of *T. catigua* showed DPPH scavenger activity (Table 2). The most potent effect was found for the hydroalcoholic extract (EC_50_ = 43 μg/ml) with potency similar to rutin, the positive control.

**Table 2:** DPPH radical scavenging and acetylcholinesterase inhibition by different extracts of *Trichilia catigua*

Extract or positive control	DPPH scavenging EC_50_ (μg/mL)	AChE inhibition IC_50_ (μg/mL)
Hexane	53 (28–136)	346 (270–502)
Chloroform	60 (35–145)	313 (193–1128)
Hydroalcoholic	43 (7–210)	142 (112–196)
Aqueous	52 (24–150)	315 (230–527)
Rutin	44 (9–216)	–
Rivastigmine	–	18 (0–136)

The EC_50_, IC_50_ and 95% confidence interval (in parenthesis) were calculated by linear regression using the mean of 3–4 assays for each concentration

### Acetylcholinesterase activity

The extracts of *T. catigua* showed in vitro AChE inhibitory activity, with the hydroalcoholic extract being the most potent with IC_50_ = 142 μg/mL (Table 2). Rivastigmine, the positive control, presented IC_50_ = 18 μg/mL.

### Motor activity

**Fig. 1:**
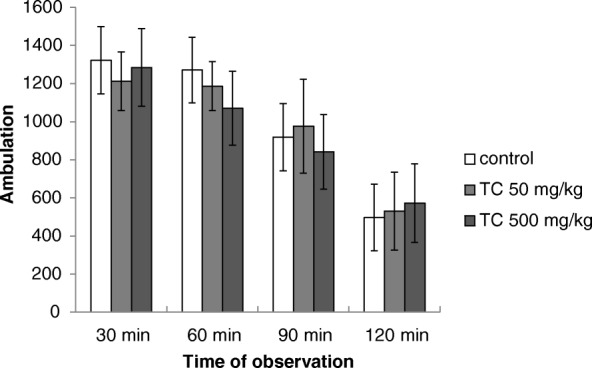
Spontaneous locomotor activity of mice treated acutely with *Trichilia catigua* (TC) hydroalcoholic extract at doses of 50 and 500 mg/kg (p.o.). The columns and bars represent the means ± SEM (*n* = 10). ANOVA, n.s

The acute treatment of mice with hydroalcoholic extract of *T. catigua* (50 and 500 mg/kg, p.o.) did not alter the locomotor activity (*p* > 0.05) during the 120 min of observation (Fig. 1). Similarly, the treatment with the same doses did not alter the motor coordination on rotarod (*p* > 0.05, Additional file 2), indicating that these doses do not disturb the animals’ motor activity.

### Stress by immobilization and cold

The stress by immobilization and cold was effective to induce increased levels of ACTH [F(3,35) = 40.05; *p* < 0.001] and corticosterone [F(3,35) = 103.81; *p* < 0.001] when comparing the stressed and non-stressed control groups (Table 3). Similarly, the stress induced gastric ulceration [F(3,35) = 8.10 (index) and 22.11 (degree); *p* < 0.001], as well as thymus and spleen atrophy [F(3,35) = 7.29 (thymus) and 30.02 (spleen); *p* < 0.001] (Table 3). However, we did not observe alteration on adrenals weight for the stressed control group. The oral treatment of rats with *T. catigua* at doses of 25 and 250 mg/kg was not able to prevent the alterations induced by cold immobilization stress. Moreover, the adrenals weight of rats treated with the dose 250 mg/kg was statistically higher (*p* < 0.05) than that of the stressed control group, indicating a tissue hypertrophy.

**Table 3:** Effect of treatment of rats with *Trichilia catigua* (TC) hydroalcoholic extract (25 and 250 mg/kg, p.o.) for 14 days on degree and index of ulceration induced by stress; on adrenal, thymus and spleen weights; and on ACTH and corticosterone plasmatic levels. The data express the mean ± SEM (*n* = 9–10)

Group	Degree of ulceration	Index of ulceration	Adrenal weight (mg)	Thymus weight (mg)	Spleen weight (mg)	ACTH (pg/mL)	Corticosterone (μg/dL)
control	3.2 ± 0.3	15.3 ± 3.1	54.4 ± 3.8	326.8 ± 21.1	809.8 ± 48.3	812 ± 31	980 ± 62
non-stressed	0.3 ± 0.2*	0.6 ± 0.3*	52.8 ± 2.6	434.2 ± 22.9*	1470.1 ± 72.6*	71 ± 27*	88 ± 35*
TC 25 mg/kg	3.7 ± 0.4^#^	19.4 ± 4.4^#^	60.1 ± 2.0	286.0 ± 29.0^#^	840.6 ± 49.5^#^	735 ± 70^#^	1012 ± 44^#^
TC 250 mg/kg	3.3 ± 0.4^#^	13.2 ± 2.3^#^	62.3 ± 2.7^#^	301.3 ± 26.4^#^	791.3 ± 66.0^#^	833 ± 84^#^	1080 ± 40^#^

(*) *p* < 0.05: statistically different of control (stressed) group; (#) *p* < 0.05: statistically different of non-stressed group. ANOVA followed by Duncan

### Forced treadmill exercise

**Fig. 2:**
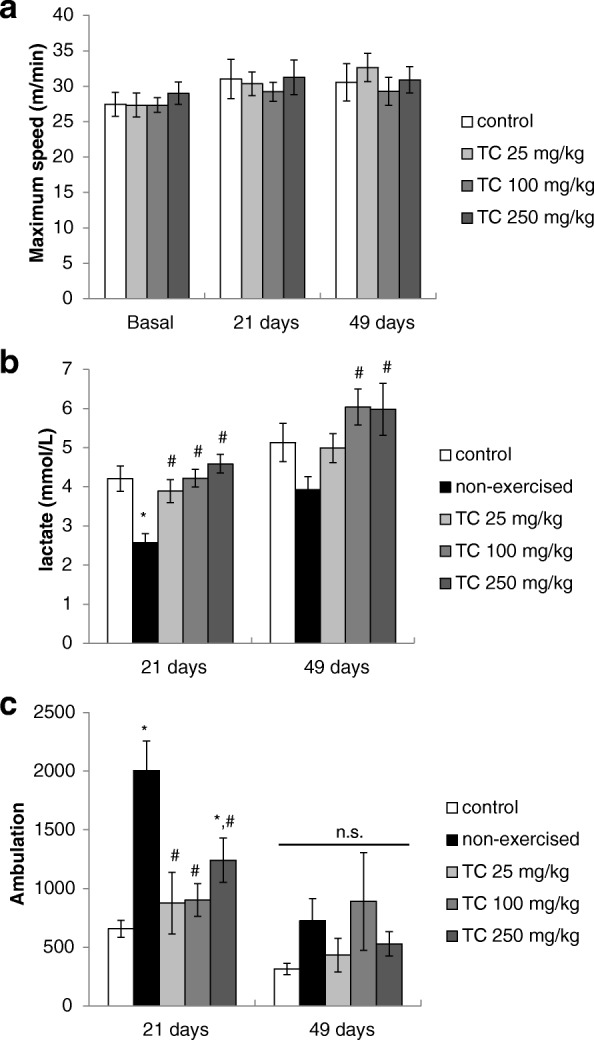
**a** Fatigue time (maximum speed), **b** plasmatic lactate level and (**c**) spontaneous locomotor activity of mice chronically treated with *Trichilia catigua* (TC) hydroalcoholic extract (25, 100 and 250 mg/kg, p.o.) and subjected to the forced exercise on treadmill. The columns and bars express the mean ± SEM (n = 8–10). (*) *p* < 0.05: statistically different of control exercised group; (#) *p* < 0.05: statistically different of non-exercised control group. ANOVA followed by Duncan

Mice were submitted to the forced exercise on treadmill before (basal) and after 21 and 49 days of treatment (3rd and 7th week) with the hydroalcoholic extract of *T. catigua*. There was no difference in fatigue time among the groups (*p* > 0.05) in the three time points (Fig. 2a). The plasmatic level of lactate after the exercise was increased [F(4,40) = 9.05; *p* < 0.001 at day 21] compared to that of mice not submitted to the treadmill (non-exercised control), but no difference was observed among the control and the groups treated with *T. catigua* (Fig. 2b).

The spontaneous locomotor activity of non-exercised mice was higher than other groups on the test carried out at 21 days of treatment [F(4,40) = 10.65; *p* < 0.001], but there was no difference among the groups when the same procedure was repeated after 49 days of treatment [F(4,39) = 1.09; *p* > 0.05]. On the other hand, the group treated with 250 mg/kg of *T. catigua* also differed from the exercised control at 21 days, showing increased locomotor activity (*p* < 0.05) after the fatigue protocol (Fig. 2c).

**Fig. 3:**
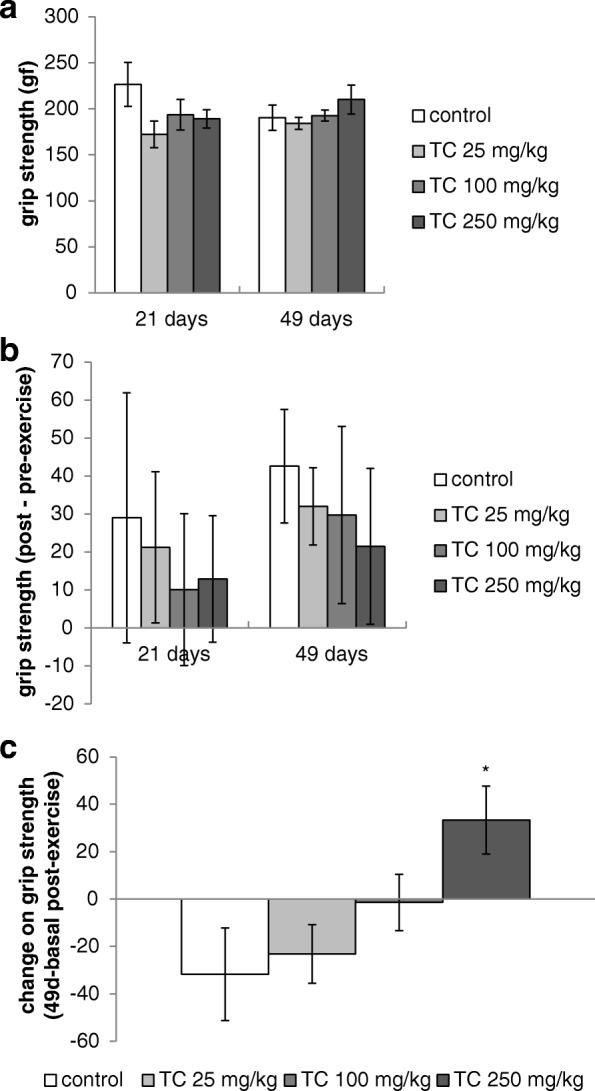
**a** Post exercise grip strength, **b** difference of grip strength (before - post-exercise) and (**c**) performance change (post-exercise grip strength 49d - basal) of mice chronically treated with *Trichilia catigua* (TC) hydroalcoholic extract (25, 100 and 250 mg/kg, p.o.) and subjected to the forced exercise on treadmill. The columns and bars express the mean ± SEM (*n* = 8–10). (*) *p* < 0.05: statistically different of control group. ANOVA followed by Duncan

The evaluation of mice grip strength after the fatigue shows that there was no difference among the groups (*p* > 0.05) in the test carried out at 21 or 49 days of treatment (Fig. 3a). The impairment of grip strength induced by exhaustion (difference on grip strength before and after the exercise) also did not change (Fig. 3b). On the other hand, when we compared the post-exercise performance in different moments, we observed that the group treated with *T. catigua* at dose of 250 mg/kg presented better performance [F(3,30) = 3.61; *p* < 0.05] than the other groups (difference on grip strength after fatigue between days 49 and basal) meaning that the animals recovered faster from the exercise on day 49 if compared with the basal evaluation (Fig. 3c).

### Classical fear conditioning

**Fig. 4:**
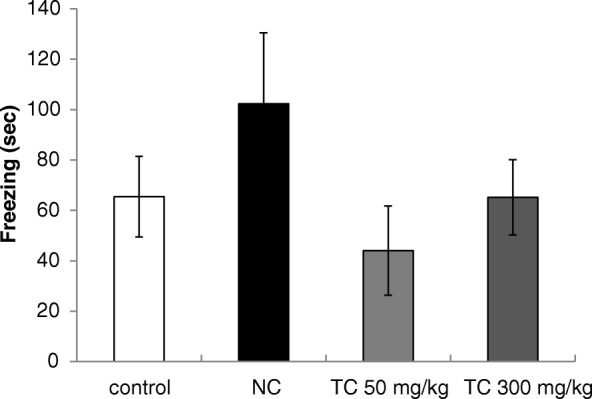
Effect of treatment with *Trichilia catigua* (TC) hydroalcoholic extract (50 and 300 mg/kg, p.o.) for 21 days on the amnesia induced by scopolamine (2 mg/kg, i.p.) on classical fear conditioning in mice. NC = negative control (did not receive scopolamine). The columns and bars express the mean ± SEM (*n* = 9–10). ANOVA, n.s

Although the pre-treatment of mice with scopolamine reduced the freezing time by 40.9% compared with control mice that did not receive scopolamine, there was no statistically significant difference among the groups [F(3,35) = 1.45; *p* > 0.05], possibly due the high variability observed. In spite of that, the freezing time of mice treated with *T. catigua* at doses of 50 and 300 mg/kg (p.o.) was similar to that of the scopolamine control group, indicating that the pre-treatment with the extract did not improve the mice memory (Fig. 4).

## Discussion

### Phytochemical analysis

The phytochemical composition of *Trichilia catigua* barks is well described in the literature. The flavalignans (flavanols substituted with phenylpropanoids) cinchonains IIa, Ia and Ib and proanthocyanidins were isolated in extracts obtained from barks of *T. catigua* [8–10]. In our study, we intended to identify the compounds extracted from the barks of *T. catigua* with different solvents in order to correlate the phytochemical profile with the in vitro activities. All extracts exhibited antioxidant and anticholinesterase activities, but the hydroalcoholic extract was the most active, which is consistent with the high reactivity of hydroxyl and carbonyl groups contained in their constituents.

Compound **1**, found in chloroform, hydroalcoholic and aqueous extract exhibited deprotonated molecule at m/z 341, which after MS/MS experiments produced base peak ion at m/z 179 (deprotonated caffeic acid). In high-resolution q-Tof mass spectrometer, the HR-ESI molecular ion [M – H]^−^ was detected at m/z 341.0909, which produced a fragmental cleavage at m/z 179.0463. Based on MS data reported by Gouveia and Castilho [30], compound **1** was assigned as 6-O-caffeoyl glucoside. For compound **6**, the HR-ESI molecular ion [M – H]^−^ was detected at m/z 353.0692, which produced a fragmental cleavage at *m/z* 191.0454. Compound 6, found only in aqueous extract, was assigned as 3-O-cafeoylquinic acid.

Exact mass can distinguish isobar molecules, which exhibit the same integer mass but different molecular formula. Isomeric structures, compounds with the same atoms, but different arrangements, cannot be separated by exact mass [31]. Compounds **7** and **12** were detected in the four extracts and could be distinguished through elution order. They exhibited the same [M − H]^−^ signal at m/z 451 in negative ESI-MS of low resolution. After MS/MS experiments produced base peak at m/z 341 (Table 1), corresponding to the loss of 110 Da, which was attributed to 3,4-dihydroxyphenyl moiety (C_6_H_6_O_2_), characteristic of cinchonains [17, 18]. Cinchonains Ia and Ib are isomers, that possess molecular formula C_24_H_20_O_9_, average mass – 452.410 g/mol and monoisotopic mass – 452.1107 g/mol. The molecular formula for compounds **7** and **12** were determined from the HR-ESI-MS molecular ion [M − H]^−^ detected at m/z 451.0788 (C_24_H_20_O_9_). The fragmental cleavage profile from the HR-ESI-MS spectrum for compound **7**, which produced base peak at m/z 341.0481, was very similar to that produced for compound **12**, providing further evidence that these compounds are isomers with the same skeleton, but different arrangements. The peak m/z 903.1652 corresponds to the dimmer of cinchonains (Figs. 7 and 12 of Additional file 1). Based on HR-ESI-MS spectra and also MS data reported by Fasciotti et al. [17] and Gu et al. [18], compounds **7** and **12** were assigned as cinchonain Ia and cinchonain Ib, respectively.

Compound **4** detected in the four extracts exhibited [M − H]^−^ signal at m/z 739, which after MS/MS experiments exhibited base peak ion at m/z 587 and a fragment ion at m/z 451. According to MS data reported by Resende et al. [8] and Fasciotti et al. [17] compound **4** was identified as cinchonain IIa. Compounds **9** and **10** detected only in chloroform extract exhibited the same deprotonated molecule at m/z 901. As can be seen in Table 1, the MS/MS spectrum of compound **9** exhibited base peak at m/z 791 derived from the loss of 3,4-dihydroxyphenyl moiety, characteristic of cinchonains. The MS/MS spectrum of compound **10** produced base base peak at m/z 597, derived from the neutral loss of (C_16_O_6_H_16_) 304 Da, but also produced fragments ions characteristic of cinchonains at m/z 791, m/z 451 and 341 [17, 18]. Compound **9** was tentatively identified as cinchonain IIa glucoside and compound **10** as cinchonain IIb glucoside. Curiously, cinchonain Ia, Ib, cinchonain IIa and cinchonain IIa glucoside were also found in bark from *Erythroxylum vaccinifolium* Mart., a medicinal plant also known as catuaba in the northeast of Brazil [32].

Compounds **8** and **11** detected in chloroform, hydroalcoholic and aqueous extract, showed the same [M − H]^−^ signal at m/z 613. The MS/MS spectrum of compound **8** produced base peak at m/z 503 and fragment ion at m/z 393, both derived from the neutral loss of 3,4-dihydroxyphenyl moiety (110 Da), which indicated the presence of two dihydroxyphenyl groups. The fragment ion at m/z 451 was derived from the neutral loss of C_9_H_6_O_3_, while the fragment ion at m/z 341 was derived from the neutral loss of C_6_H_6_O_2_ + C_9_H_6_O_3_ (Table 1). Since the fragment ions of compound **8** match the ones reported by Gu et al. [18], it was assigned as a bis-(3,4-dihydroxyphenylpropanoid)-substituted catechin. The ESI-MS spectrum of compound **11,** also exhibited a base peak at m/z 503 resulting from the neutral loss of 3,4-dihydroxyphenyl moiety (110 Da), but the relative intensities of fragments ions at m/z 451 and m/z 341 are very low. According to mass spectral database HMDB [24], compound **11** was tentatively assigned as cinchonain Id-7- glucoside.

Proanthocyanidins are polymeric flavonoids based on flavan-3-ols (oligomers of catechin and/or epicatechin and their gallic acid esters). Compounds **2** and **3** also exhibited UV maximum absorption at 280 nm and are proposed to be type B dimmer proanthocyanidins. These compounds were detected only in hydroalcoholic extract and exhibited [M − H]^−^ signals at m/z 723 and 577, respectively. The MS/MS spectra of compounds 2 and 3 produced fragment ions at m/z 425, 407 and 289 (Table 1) characteristic for procyanidin B-type dimmers and a fragment ion at m/z 289 (catechin). Retro-Diels-Alder reaction of the heterocyclic ring system of the flavan-3-ol subunits gave rise to a fragment of m/z 425. The ion at m/z 425 eliminates water, probably from ring C at position C3/C4, resulting in a fragment ion of m/z 407 [19, 20]. Based on MS data reported by Kicel et al. [20], compound **3** was identified as type B dimmer of proanthocyanidin (epi) catechin - (epi) catechin, known as procyanidin B2. The MS/MS spectrum of compound **2** also exhibited abundant fragment ion at m/z 433, resulting from the loss of 290 Da (catechin). Based on mass spectral database [24], compound **2** was tentatively identified as procyanidin B2–8-C-rhamnoside. Compound **5** detected only in chloroform extract, exhibited [M − H]^−^ signal at m/z 467, which after MS/MS experiments produced base peak at m/z 449, resulting from the loss of water. Compound **5** was tentatively assigned as apocynin E, which was also detected by Resende et al. [8] in *T. catigua* bark.

The presence of procyanidins and cinchonains in *T. catigua* bark was corroborated by the studies of Truiti et al. [16] and Resende et al. [8] that obtained a similar chemical profile. On the other hand, flavonoids rutin and quercetin identified by Kamdem et al. [15] and catiguanin A and catiguanin B (phenylpropanoid-substituted epicatechins) isolated from the bark of *T. catigua* by Tang et al. [33] were not present in our extracts in detectable amounts. However, as far as we know, a bis-(3,4-dihydroxyphenylpropanoid)-substituted catechin (**8**), cinchonain Id-7- glucoside (**11**) and cinchonain II glucosides (**9** and **10**) were detected for the first time in the species. This difference in chemical composition could be explained leading in consideration that the chemical constituents can vary in their structure and concentration depending on the region and season of collection, genetic variability, as well as the extraction method.

### Biological activity

Several studies have shown the antioxidant activity of different catuaba (*T. catigua*) extracts [8, 33–35]. The data of our study confirm the antioxidant effect of *T. catigua* on DPPH assay for the four extracts analyzed. The most potent effect was achieved with the hydroalcoholic extract (EC_50_ = 43 μg/ml), with potency similar to rutin (EC_50_ = 44 μg/ml), the positive control, followed by aqueous, hexane and chloroform extracts. Lonni et al. [35] compared the antioxidant capacity (DPPH assay) of *T. catigua* extracted with different solvents and found the best result with ethanol, followed by acetone, water and methanol. In other study, Kamdem et al. [34] found that the content of total phenolics was higher in ethyl acetate extract, but the best effect on DPPH assay was obtained for the ethanolic extract. Using compounds isolated from *T. catigua* bark, Resende et al. [8] observed the most potent antioxidant activity with procyanidin C1, cinchonain IIb and cinchonain IIa, while Tang et al. [33] found the best results with the cinchonains Id, Ic and Ib. In our study, the hydroalcoholic extract containing cinchonains and procyanidins also exhibited the most potent antioxidant activity.

The neuroprotective activity of *T. catigua* is mainly attributed to its antioxidant activity. The 70% ethanolic extract of catuaba at concentrations from 10 to 100 μg/ml protected hippocampal neurons in vitro from oxidative stress and increased the survival after ischemia and reperfusion [15] or in the presence of hydrogen peroxide, sodium nitroprusside and nitropropionic acid [6]. The crude extract (acetone:water 7:3) and its semipurified fraction (partitioned with ethyl acetate), rich in epicatechin and procyanidin B2, were administered to mice in doses of 200 to 800 mg/kg for 7 days before the animals were submitted to a bilateral occlusion of the carotid. The treatment improved the performance of the animals in the Morris water-maze and protected hippocampal neurons [16]. These effects were mainly assigned to flavonoids and polyphenols present in these extracts, due to their antioxidant activity.

Other effects, as antinociceptive and antidepressant-like effect, seem to be related to a dopaminergic action [12, 13]. Neurochemical studies showed that the ethanolic extract of *T. catigua* inhibited dopamine and serotonin uptake and increased the release of these neurotransmitters, with more potent activity to dopamine. The antidepressant-like effect was evaluated in animals treated with doses of 200 and 400 mg/kg in the forced swimming test and tail suspension test. The extract induced antidepressant-like effect, which was blocked by haloperidol and chlorpromazine, anti-dopaminergic agents [13]. Another study using the ethyl acetate fraction of *T. catigua* showed antidepressant-like effect and increased cellular proliferation in the hippocampus [14].

The central cholinergic system is involved in the regulation of many cognitive functions and cholinergic alterations that occur during aging are associated with learning and memory deficits. Acetylcholinesterase hydrolyzes the acetylcholine released on central nervous system synapses regulating its concentration and effect. However, there is a progressive loss of cholinergic neurons that innervate hippocampus and the neocortex in Alzheimer’s disease and some other dementias resulting on cholinergic hypofunction. AChE inhibitors are used clinically on the treatment of Alzheimer’s disease, because they increase the availability of acetylcholine present in cholinergic synapses, enhancing the cholinergic functions. Drugs as rivastigmine (used as positive control in our study), galantamine and huperzine A (active principles isolated from medicinal plants) are AChE inhibitors employed in the treatment of Alzheimer’s disease [36].

In the current study, the effect of *T. catigua* extracts on cholinergic system was evaluated for the first time. All extracts tested inhibited the activity of acetylcholinesterase in vitro, and the most potent effect was obtained for the hydroalcoholic extract (IC_50_ = 142 μg/ml), followed by chloroform, aqueous and hexane extracts, with IC_50_ ranging from 313 to 346 μg/ml. The inhibition of AChE demonstrated for the four extracts may be due to the presence of high contents of cinchonains IIa, Ia and Ib, which are flavalignans - flavanols substituted with phenylpropanoids. Flavonoids that possess a free OH-group at C3 position showed major activity when compared to their C3 − OH glycosylated counterparts and those having no C3 − OH group, such as luteolin and apigenin [37, 38]. The major inhibition observed for the hydroalcoholic extract can be explained by the presence of procyanidins B2 found only in this extract. Proanthocyanidins exhibited a potent role in enhancing cognition in older rats, which was attributed to an increase in the acetylcholine concentration with a moderate reduction in AChE activity [39]. Proanthocyanidins exhibited ameliorative effects on learning and memory impairment of mice in scopolamine-induced amnesia test, showing protection against memory deficit [40].

The anticholinesterase effect found in our study can support the promnesic effect observed by Chassot et al. [5] for the crude extract and ethyl-acetate fraction of *T. catigua*. However, the hydroalcoholic extract of catuaba in doses of 50 and 300 mg/kg in our study did not promote memory improvement in mice treated with scopolamine, a competitive antagonist of muscarinic receptors. The inhibition of AChE causes an increase of concentration and time of acetylcholine on synaptic cleft, facilitating the cholinergic transmission. However, it is not possible to know in this study whether the in vitro anticholinesterase effect is also present in vivo. Or perhaps, the increase in acetylcholine concentration may not be enough to displace the scopolamine from the receptor and avoid its amnesic effect.

Kamdem et al. [15] discuss that *T. catigua* ethanolic extract seems to have preventive, but not curative effect on experimental ischemia, since the in vitro treatment of hippocampal slices after the protocol of ischemia and reperfusion did not protect the neurons. This prophylactic profile corroborates with the expected effect of an adaptogen, which is used chronically to avoid or diminish damages from stress and aging. In fact, the folk use of catuaba is similar to what we would expect for a typical adaptogen: the plant is used chronically to prevention and treatment of neurasthenia, fatigue, stress, impotence and memory deficits [1].

This is the first study evaluating the effect of *T. catigua* on stress and fatigue. We employed the hydroalcoholic extract of catuaba, which corresponds to the form popularly used and that showed the best results in our in vitro tests. The doses employed were comparable with those of previous in vivo studies and they did not interfere with the locomotor activity and motor coordination on rotarod, suggesting they were safe. The treatment with catuaba at doses of 25 and 250 mg/kg p.o. (starting 7 days before the repeated stress protocol) did not protect the animals from ulceration, neither prevented corticosterone and ACTH increase or thymus and spleen atrophy induced by stress. Adaptogens can lightly raise the basal level of corticosteroids, nevertheless adaptogens prevent the overwhelming increase of cortisol induced by stress [41]. The protocol of cold and immobilization causes an intense stress on the animal, seeing that the levels of ACTH and corticosterone increased tenfold in control-stressed rats when compared with non-stressed controls. Catuaba is widely used against fatigue and stress, but as far as we known, it is not used to treat or prevent gastric ulcers.

In order to evaluate whether *T. catigua* has an antifatigue effect, mice were chronically treated with hydroalcoholic extract at doses of 25, 100 and 250 mg/kg (p.o.) and submitted to forced exercise on a treadmill in three phases: before the treatment (basal performance) and after 21 and 49 days of treatment. The administration of catuaba did not alter the fatigue time, nor the lactate levels measured immediately after the exercise. However, mice treated with the highest dose showed increased spontaneous locomotor activity after the forced exercise on the 21th day. This result suggests that the treatment with catuaba may decrease the recovery time after an exhaustion protocol. Moreover, catuaba treatment for 49 days at the highest dose was able to diminish the impact of the forced exercise on the animals’ strength since the impairment on grip strength after the exercise was shortened at day 49 compared with the basal performance (difference on grip strength after fatigue between days 49 and basal). Even modest, these results suggest that the hydroalcoholic extract of catuaba may have beneficial effects on fatigue, at least shortening the recovery time after exhaustion. Stress-protective and antifatigue effects have been described for some adaptogens, as *Rhodiola rosea* L., *Eleutherococcus senticosus* (Rupr. & Maxim.) Maxim. and *Panax ginseng* C.A. Meyer and several clinical trials were already conducted [41]. The importance of antioxidants on physical exercise and to prolong endurance and reduce fatigue has been evaluated. An extract of *Polygonatum altelobatum* Hayata rich in polyphenols and polysaccharides increased the endurance running time to exhaustion and the antioxidant ability in rats’ blood [42]. A supplementation with *Chaenomeles speciosa* (Sweet) Nakai fruit prolonged the exhaustive swimming time of rats and raised antioxidant enzymes levels, possibly by modulating the Nrf2 pathway [43].

*Panax ginseng* and other adaptogens are chronically used for several purposes – to increase stress resistance and physical capacity, to improve memory and other cognitive functions and as neuroprotective agents [44]. Ginseng acts by multiple mechanisms of action: it reduces the oxidative stress and excitotoxicity, modulates cholinergic neurotransmission, and increases dopamine and noradrenaline in the cerebral cortex [44]. It is likely that both the acetylcholinesterase inhibition and the antioxidant effect of *T. catigua* may contribute to its neuroprotective and pro-cognitive effects, as well as its dopaminergic and serotonergic effects are important for its antinociceptive and antidepressant effects. The antioxidant effect of different extracts or isolated constituents of catuaba was well evaluated. Several studies confirm that ethanolic or hydroalcoholic extracts of catuaba seems to have the most potent antioxidant effect [6, 33–35], but the proportion of water and ethanol can be better explored. Another alternative should be the use of special extracts prepared by extraction with different solvents, as suggested by Lonni et al. [35].

## Conclusions

In brief, we confirmed the presence of cinchonains and procyanidins in *T. catigua* and found the best antioxidant and anticholinesterase activity for the hydroalcoholic extract. This extract did not avoid the damages induced by stress and did not prevent the amnesia induced by scopolamine, but had a mild protective effect on forced exercise and fatigue. These data suggest the hydroalcoholic extract as the most suitable for plant extraction and partially support the folk use of *T. catigua* as antifatigue drug.

## Additional files

Additional file 1: HPLC-ESI-MS/MS spectra of the compounds 1–12. Total ion current chromatogram, ESI-MS/MS in negative mode and Q-Tof – mass spectrometry of the main compounds found in the extracts. (PDF 175 kb)

Additional file 2: Effect of acute treatment of mice with *Trichilia catigua* hydroalcoholic extract on rotarod performance. Table showing the mean ± EPM of the control and experimental groups on rotarod. (PDF 13 kb)

## Abbreviations

AChE: Acetylcholinesterase
ACTH: Adrenocorticotropic hormone
DPPH: 2,2-diphenyl-1-picryl hydrazyl
DTNB: 5,5-dithiobis-2-nitrobenzoic acid
Q-ToF: Hybrid quadrupole orthogonal acceleration time-of-flight mass spectrometer
RPHPLC-DAD-ESI-MS/MS: Reverse phase high performance liquid chromatography-diode array-electrospray ionization- mass spectra-mass spectra
