# Supplementary material for: Antioxidant, anticholinesterase and antifatigue effects of Trichilia catigua (catuaba)
Source: BMC Complement Altern Med. 2018 Jun 5;18:172. doi: 10.1186/s12906-018-2222-9 (PMC5987406; doi:10.1186/s12906-018-2222-9)
Supplement: Supplementary file 1 — HPLC-ESI-MS/MS spectra of the compounds 1–12. Total ion current chromatogram, ESI-MS/MS in negative mode and Q-Tof – mass spectrometry of the main compounds found in the extracts. (PDF 175 kb) [file 12906_2018_2222_MOESM1_ESM.pdf]

Antioxidant, anticholinesterase and antifatigue effects of *Trichilia catigua* (catuaba)

Nadini Oliveira Martins, Isabella Modelli de Brito, Sandra Syomara O. Araújo, Giuseppina Negri, Elisaldo de Araújo Carlini, Fúlvio Rieli Mendes

Supplementary Material

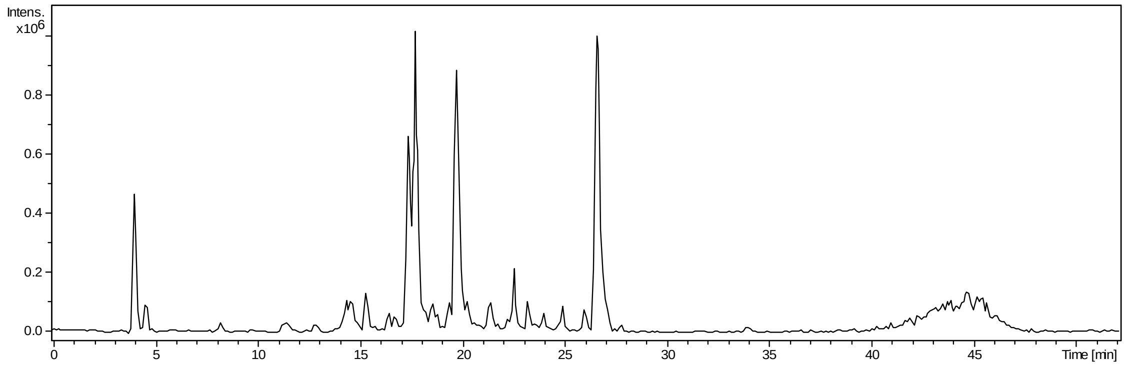

Figure 1S: Total ion current chromatogram obtained through HPLC-DAD-ESI-MS analysis of hydroethanolic extract from *T. catigua.*

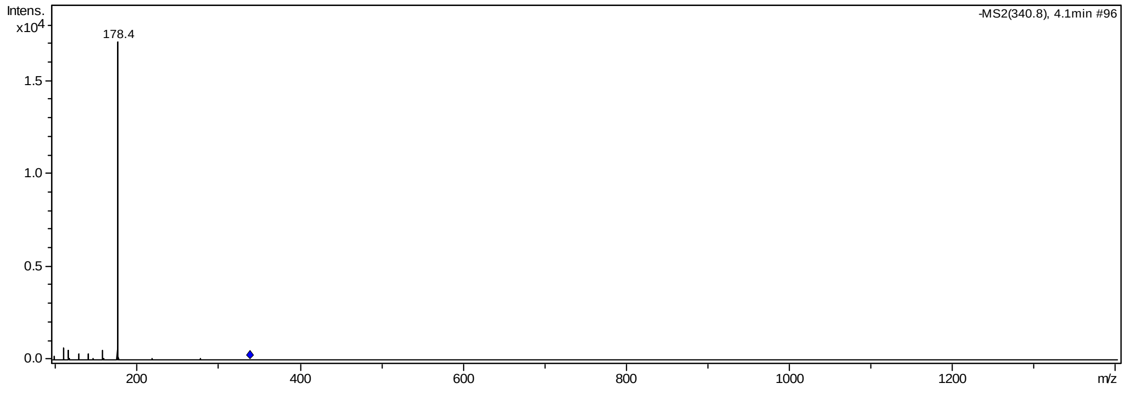

Figure 2S: ESI-MS/MS in negative mode of compound **1** - 6-O-caffeoyl glucoside.

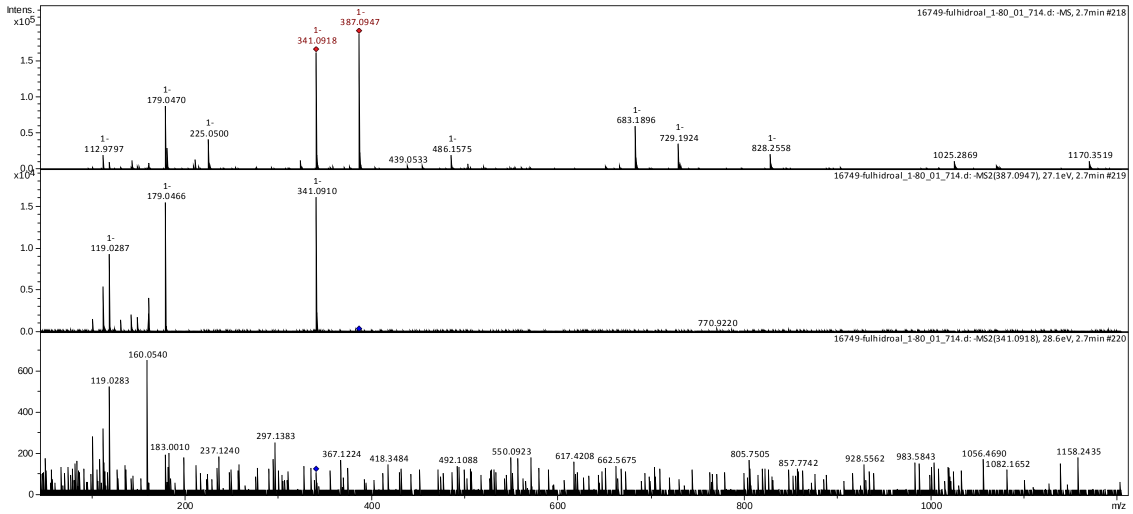

Figure 3S: Q-Tof ï mass spectrometry - MAXIS 3G ï Bruker Daltonics of compound **1** - 6-O-caffeoyl glucoside.

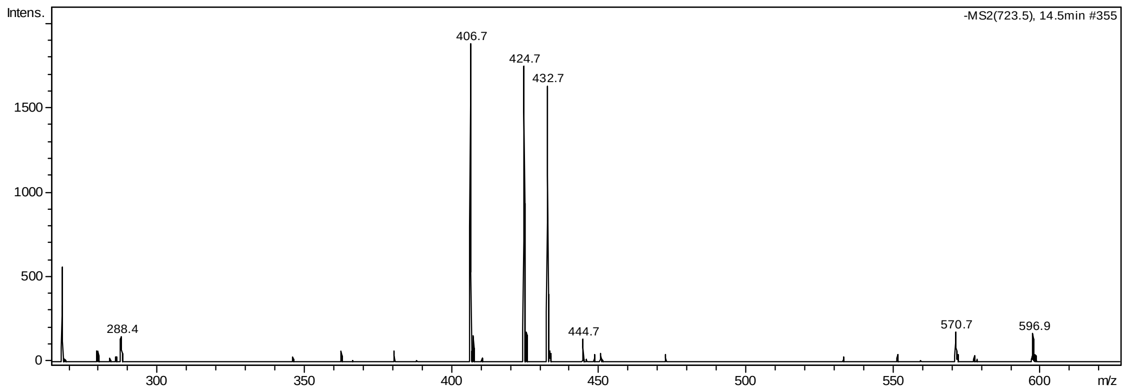

Figure 4S: ESI-MS/MS negative mode of compound **2** - Procyanidin B2-8-C-rhamnoside.

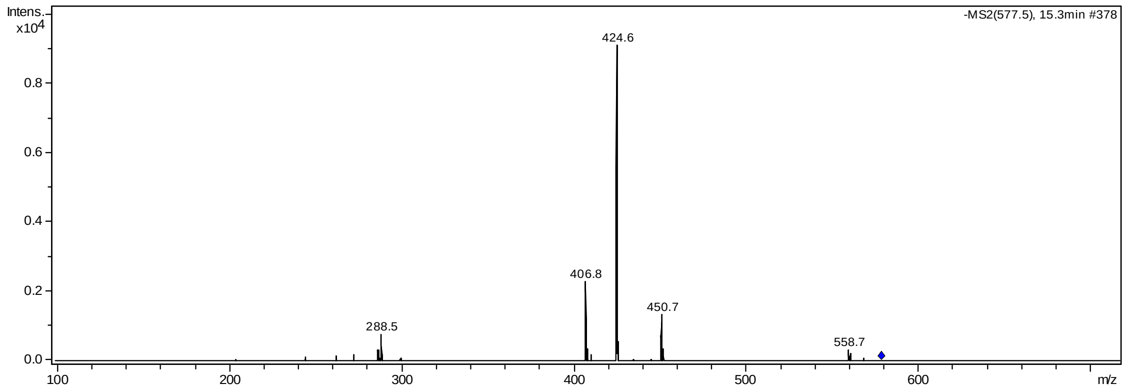

Figure 5S: ESI-MS negative mode of compound **3** - Procyanidin B2 (epi)-catechin ï (epi)-catechin.

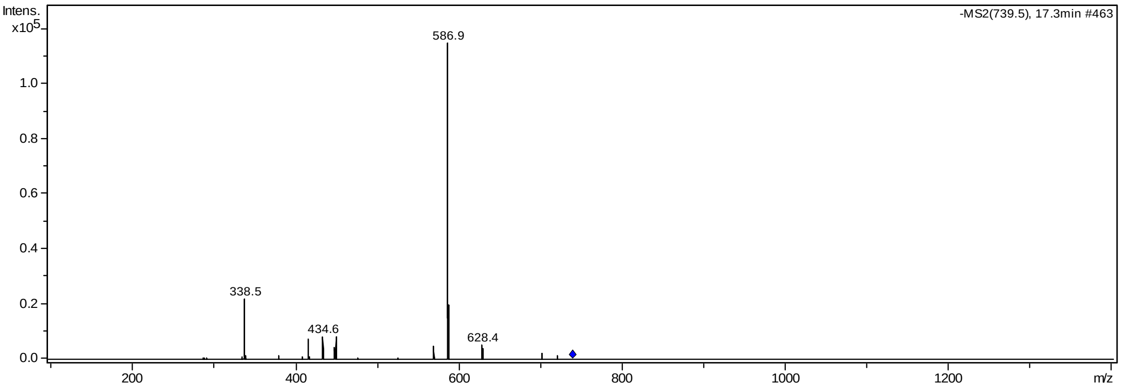

Figure 6S: ESI-MS negative mode of compound **4** - Cinchonain IIa.

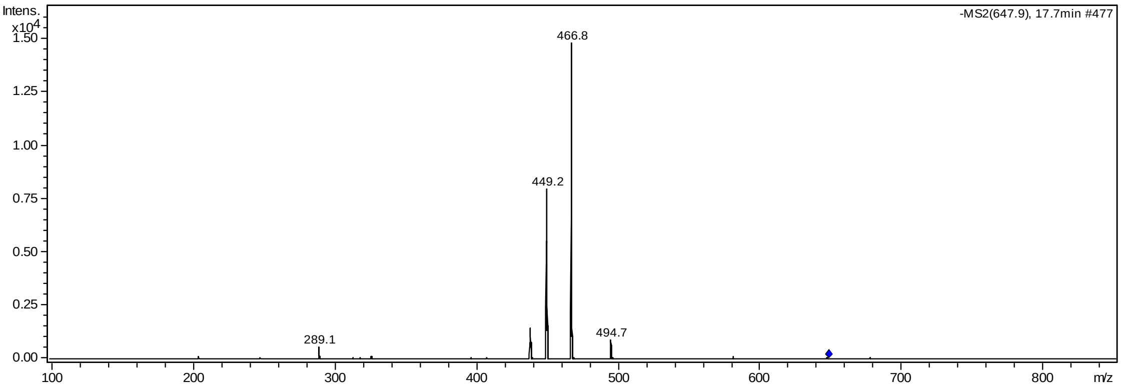

Figure 7S: ESI-MS negative mode of compound **5** ï apocynin E.

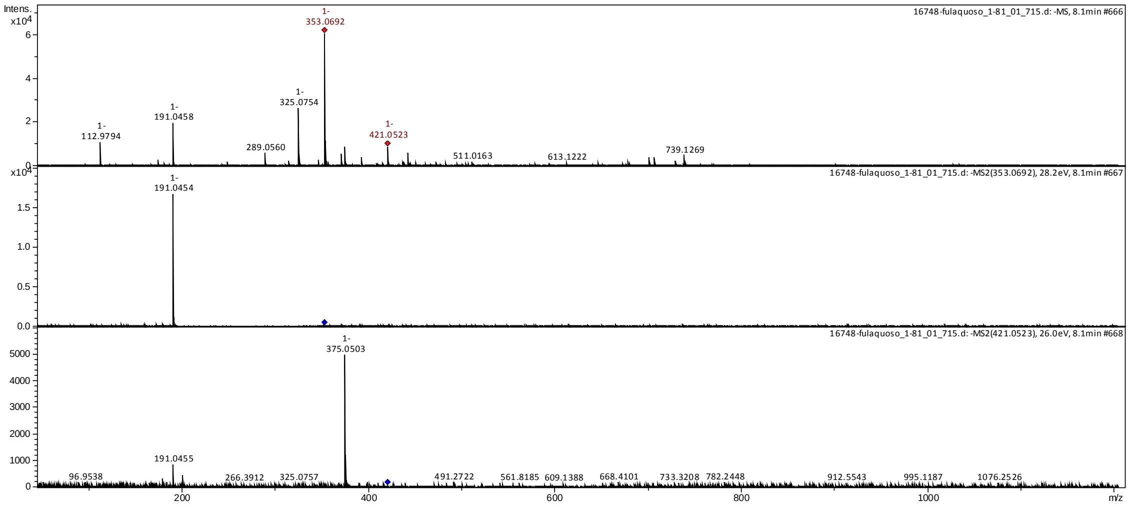

Figure 8S: Q-Tof ï mass spectrometry - MAXIS 3G ï Bruker Daltonics of compound **6** ï 3-O-caffeoylquinic acid.

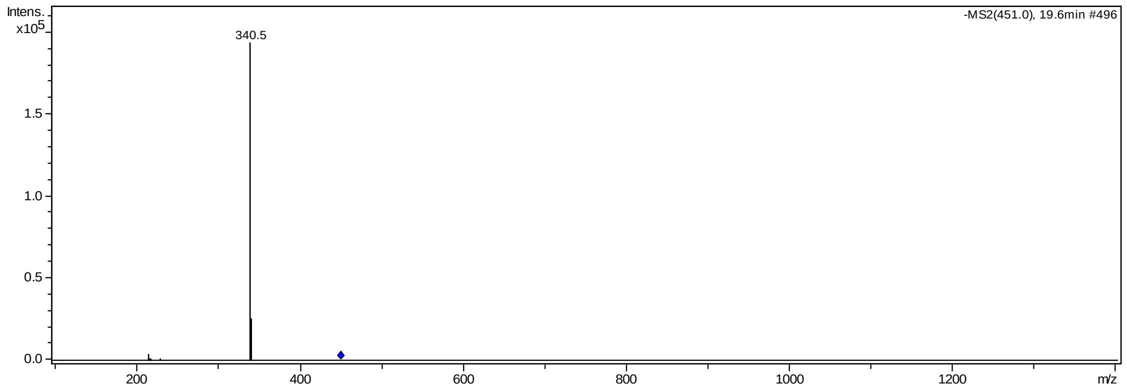

Figure 9S: ESI-MS negative mode of compound **7** - Cinchonain Ia.

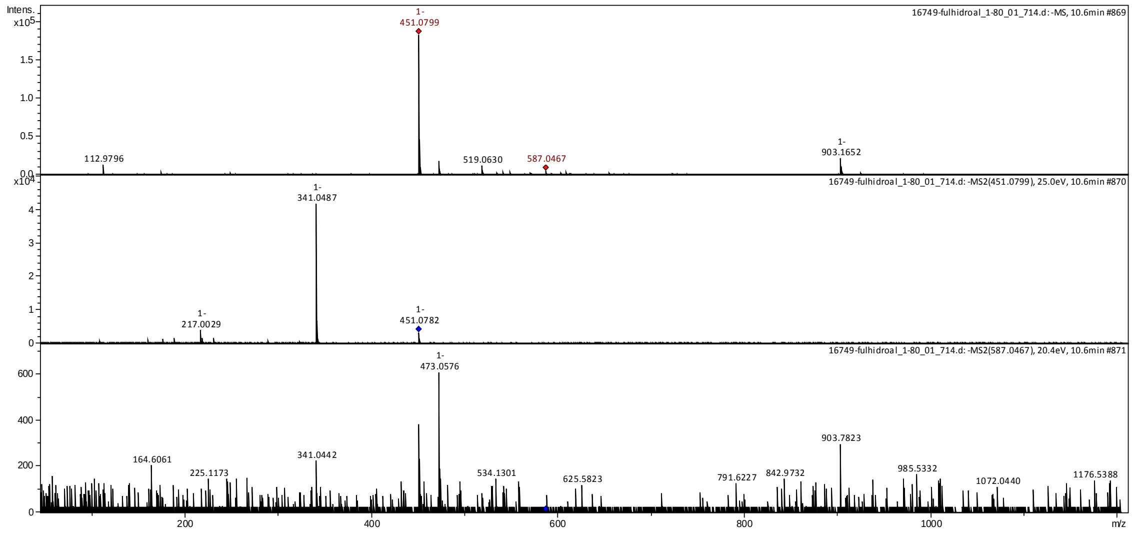

Figure 10S: Q-Tof ï mass spectrometry - MAXIS 3G ï Bruker Daltonics of compound **7**- Cinchonain Ia.

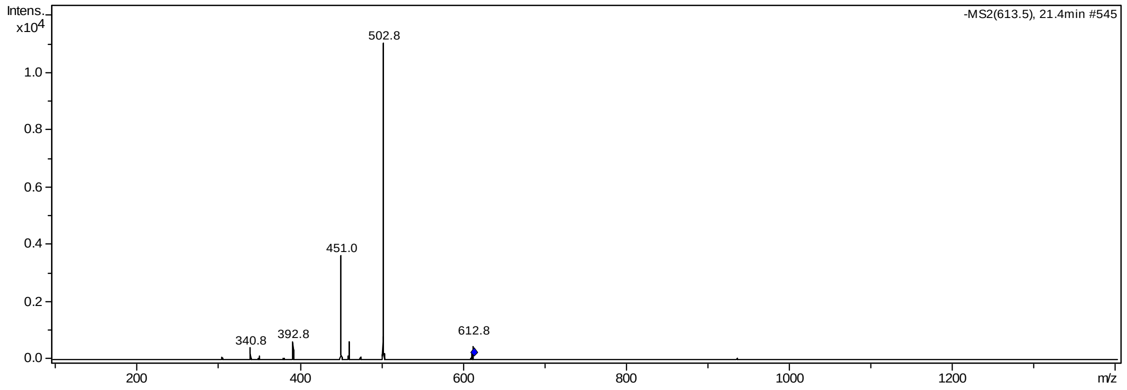

Figure 11S: ESI-MS in negative mode of compound **8** - bis-(3,4-dihydroxyphenylpropanoid)-substituted catechin**.**

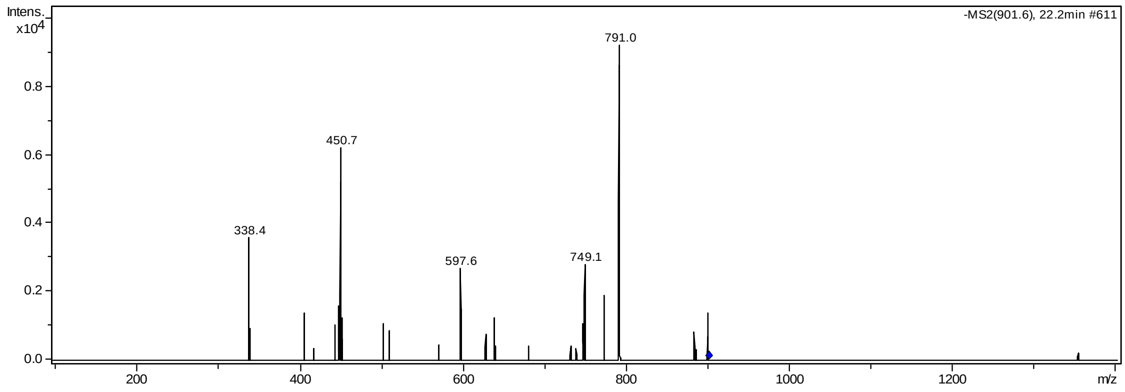

Figure 12S: ESI-MS in negative mode of compound **9** - Cinchonain IIa glucoside.

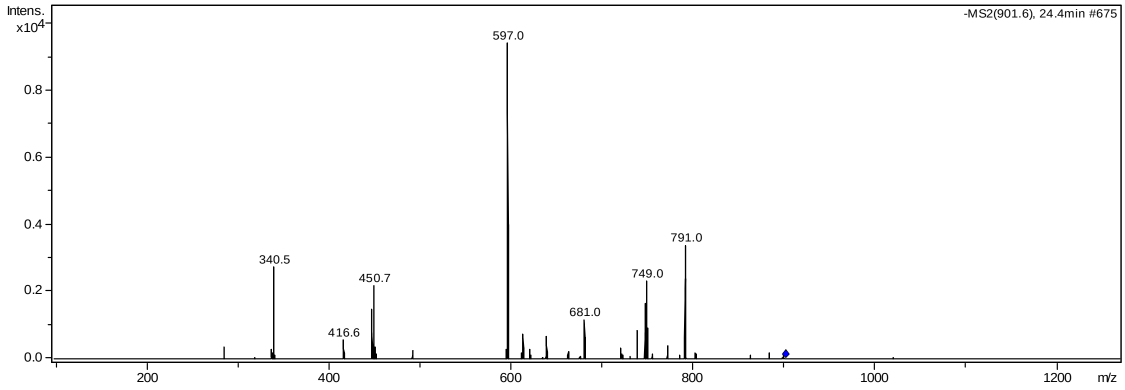

Figure 13S: ESI-MS in negative mode of compound **10** - Cinchonain IIb glucoside.

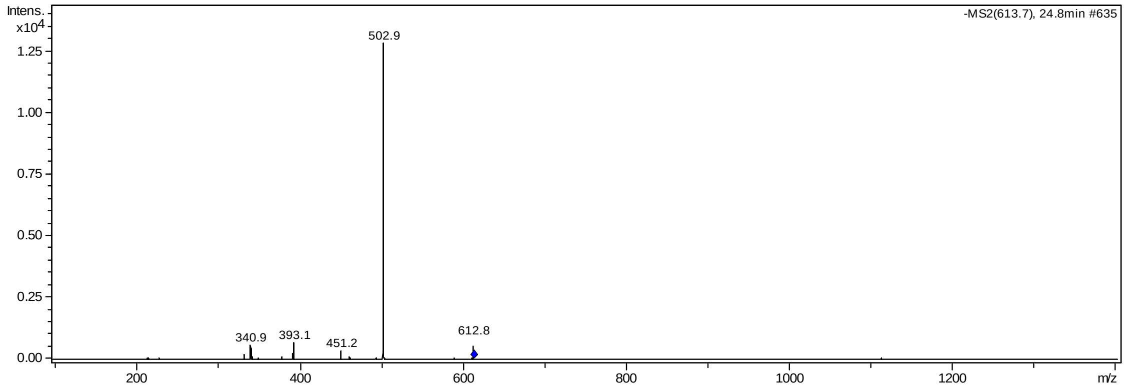

Figure 14S: ESI-MS in negative mode of compound **11** - Cinchonain Id-7- glucoside.

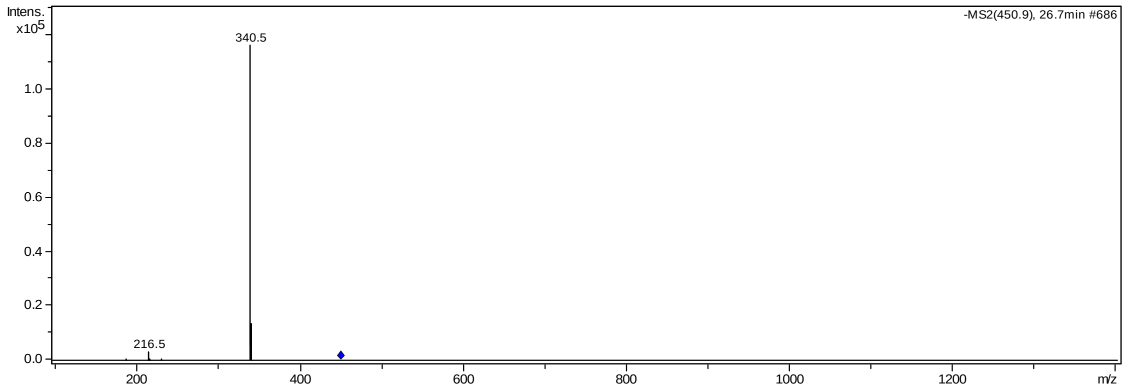

Figure 15S: ESI-MS in negative mode of compound **12** - Cinchonain Ib.

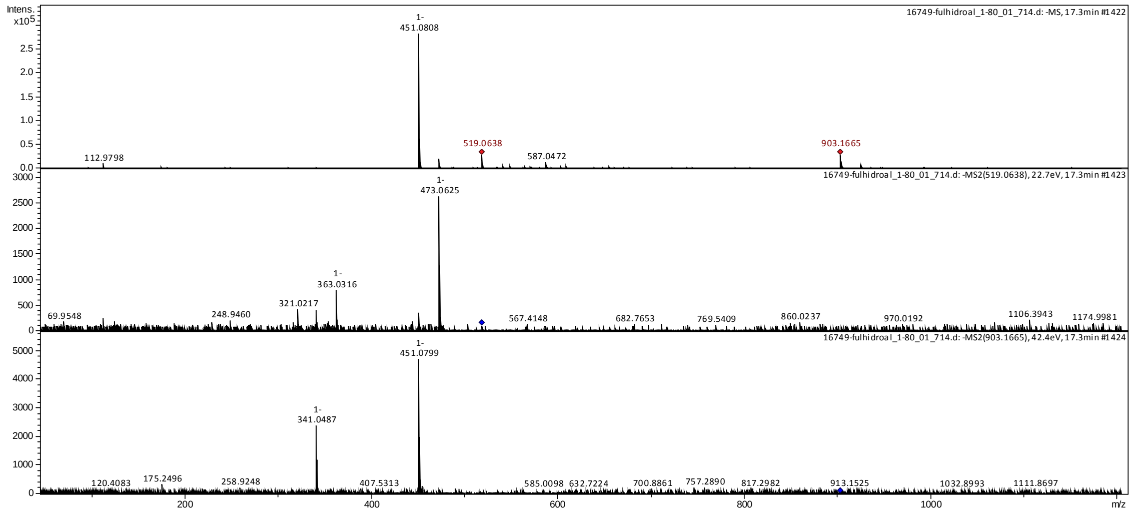

Figure 16S: Q-Tof ï mass spectrometry - MAXIS 3G ï Bruker Daltonics of compound **12** - Cinchonain IIa.
