# Supplementary material for: Antioxidant, anticholinesterase and antifatigue effects of Trichilia catigua (catuaba)
Source: BMC Complement Altern Med. 2018 Jun 5;18:172. doi: 10.1186/s12906-018-2222-9 (PMC5987406; doi:10.1186/s12906-018-2222-9)
Supplement: Supplementary file 2 — Effect of acute treatment of mice with Trichilia catigua hydroalcoholic extract on rotarod performance. Table showing the mean ± EPM of the control and experimental groups on rotarod. (PDF 13 kb) [file 12906_2018_2222_MOESM2_ESM.pdf]

Antioxidant, anticholinesterase and antifatigue effects of *Trichilia catigua* (catuaba)

Nadini Oliveira Martins, Isabella Modelli de Brito, Sandra Syomara O. Araújo, Giuseppina Negri, Elisaldo de Araújo Carlini, Fúlvio Rieli Mendes

Supplementary Material

Table 1S: Effect of acute treatment of mice with *Trichilia catigua* hydroalcoholic extract (50 and 500 mg/kg, p.o.) on rotarod performance. The data show the means ± SEM (n = 10).

GROUP	Dose (mg/kg)	Time of observation (min)			
		Basal	30[illegible]	60[illegible]	120[illegible]
**Control**	-	58.5 ± 1.5	52.8 ± 4.1	55.4 ± 2.7	60.0 ± 0
T. catigua	50	51.5 ± 4.4	60.0 ± 0	60,0 ± 0	60,0 ± 0
T. catigua	500	45.7 ± 5.5	48.2 ± 5.5	54.8 ± 3.0	58.0 ± 2.0

n.s. Kruskal-Wallis.
